# Contribution of TRPC Channels in Neuronal Excitotoxicity Associated With Neurodegenerative Disease and Ischemic Stroke

**DOI:** 10.3389/fcell.2020.618663

**Published:** 2021-01-08

**Authors:** Jaepyo Jeon, Fan Bu, Guanghua Sun, Jin-Bin Tian, Shun-Ming Ting, Jun Li, Jaroslaw Aronowski, Lutz Birnbaumer, Marc Freichel, Michael X. Zhu

**Affiliations:** ^1^Department of Integrative Biology and Pharmacology, McGovern Medical School, The University of Texas Health Science Center at Houston, Houston, TX, United States; ^2^Department of Neurology, McGovern Medical School, The University of Texas Health Science Center at Houston, Houston, TX, United States; ^3^Institute for Biomedical Research (BIOMED UCA-CONICET), Buenos Aires, Argentina; ^4^School of Medical Sciences, Catholic University of Argentina (UCA), Buenos Aires, Argentina; ^5^Neurobiology Laboratory, National Institute of Environmental Health Sciences, Durham, NC, United States; ^6^Department of Pharmacology, Heidelberg University, Heidelberg, Germany; ^7^DZHK (German Centre for Cardiovascular Research), partner site Heidelberg/Mannheim, Heidelberg, Germany

**Keywords:** neurological disease, TRPC4 knockout, calcium, neuroprotection, neurodegeneration, neuronal death

## Abstract

The seven canonical members of transient receptor potential (TRPC) proteins form cation channels that evoke membrane depolarization and intracellular calcium concentration ([Ca^2+^]_*i*_) rise, which are not only important for regulating cell function but their deregulation can also lead to cell damage. Recent studies have implicated complex roles of TRPC channels in neurodegenerative diseases including ischemic stroke. Brain ischemia reduces oxygen and glucose supply to neurons, i.e., Oxygen and Glucose Deprivation (OGD), resulting in [Ca^2+^]_*i*_ elevation, ion dyshomeostasis, and excitotoxicity, which are also common in many forms of neurodegenerative diseases. Although ionotropic glutamate receptors, e.g., *N*-methyl-D-aspartate receptors, are well established to play roles in excitotoxicity, the contribution of metabotropic glutamate receptors and their downstream effectors, i.e., TRPC channels, should not be neglected. Here, we summarize the current findings about contributions of TRPC channels in neurodegenerative diseases, with a focus on OGD-induced neuronal death and rodent models of cerebral ischemia/reperfusion. TRPC channels play both detrimental and protective roles to neurodegeneration depending on the TRPC subtype and specific pathological conditions involved. When illustrated the mechanisms by which TRPC channels are involved in neuronal survival or death seem differ greatly, implicating diverse and complex regulation. We provide our own data showing that TRPC1/C4/C5, especially TRPC4, may be generally detrimental in OGD and cerebral ischemia/reperfusion. We propose that although TRPC channels significantly contribute to ischemic neuronal death, detailed mechanisms and specific roles of TRPC subtypes in brain injury at different stages of ischemia/reperfusion and in different brain regions need to be carefully and systematically investigated.

## Introduction

Stroke occurs when a part of the brain is deprived of oxygen and glucose. Each year, about 795,000 Americans suffer a new or recurrent stroke, making it the No. 5 cause of death and a leading cause of disability in the United States (Virani et al., 2020). In 70–80% of the cases, the precipitating cause of stroke is a blood clot that blocks the supply of oxygenated blood to a region of the brain, a situation termed ischemic stroke (Woodruff et al., 2011). The damage to neurons during ischemia is caused by a reduction of oxygen and glucose supply, i.e., oxygen and glucose deprivation (OGD) (Ying et al., 1997; Lipton, 1999).

Cell death after cerebral ischemia may result from a number of events, including acidosis, inflammation, generation of arachidonic acid, elevation in intracellular calcium concentrations ([Ca^2+^]_*i*_), loss of cellular ion homeostasis, free radical-mediated toxicity, energy failure, infiltration of leukocytes, cytokine-mediated cytotoxicity and glutamate-induced excitotoxicity (Woodruff et al., 2011). Particularly, excessive extracellular glutamate can elicit multiple neurotoxic effects. Initially, energy depletion-induced depolarization of the neuronal membrane leads to the influx of Ca^2+^ through voltage-gated Ca^2+^ channels (VGCCs), which triggers Ca^2+^-dependent glutamate release from axonal terminals of excitatory neurons (Bonde et al., 2005). In the past, much of the focus on glutamate-induced excitotoxicity had been on ionotropic glutamate receptors (iGluRs), mainly NMDA (*N*-methyl-D-aspartate) receptors and AMPA (a-amino-3-hydroxy-5-methyl-4-isoxazolepropionic acid) receptors, but more recently, metabotropic glutamate receptors (mGluRs) have also been recognized to play a crucial role in excitotoxicity (Hilton et al., 2006).

The mGluR family consists of three groups, group I-III, and 8 subtypes, mGluR1-8, of which the group I mGluRs, i.e., mGluR1 and mGluR5, are coupled to G_*q/*__11_-phospholipase C (PLC) pathway and the rest are all linked to G_*i/o*_ proteins (Swanson et al., 2005). On one hand, the activation of G_*q/*__11_-PLC pathway by glutamate through stimulation of mGluR1/5 is to enhance neuronal excitability via a number of mechanisms, such as the suppression of K^+^ channels caused by the breakdown of phosphatidylinositol 4,5-bisphosphate (PIP_2_), a phospholipid that supports the activity of many K^+^ channels (Hille et al., 2015), and the production of inositol 1,4,5-trisphosphate (IP_3_), a second messenger that acts at the IP_3_ receptors to mobilize Ca^2+^ from the endoplasmic reticulum (ER) Ca^2+^ stores (Berridge, 2009). On the other hand, the activation of G_*i/o*_-coupled group II and group III mGluRs are generally thought to inhibit neuronal excitation through, among others, activation of G protein-gated inwardly rectifying K^+^ (GIRK) channels and inhibition of VGCCs (Logothetis et al., 2015). Therefore, the excessive extracellular glutamate associated with OGD may lead to both excitatory and inhibitory effects through mGluRs depending on the abundance and types of the mGluRs and downstream signaling pathways involved.

A major class of Ca^2+^-permeable non-selective cation channels activated downstream from mGluRs is the Transient Receptor Potential Canonical (TRPC) channels. The TRPC channel family consists of 7 members, TRPC1-7, in which TRPC2 is a pseudogene in humans (Montell et al., 2002). These channels are typically activated downstream from receptors linked to PLC signaling, with G_*q/*__11_-PLCβ pathway being the most common. Thus, glutamate activation of G_*q/*__11_-coupled mGluR1/5 is likely linked to TRPC channel activation, leading to consequent membrane depolarization and [Ca^2+^]_*i*_ elevation. Ironically, despite the widespread expression of TRPCs in brain neurons (Riccio et al., 2002) and their much-longer lasting activities in response to G_*q/*__11_-PLCβ signaling than iGluRs (Hartmann et al., 2008; Riccio et al., 2009), not much is known about the contributions of TRPC channels in excitotoxicity to neurons except for a few examples. It was shown that in the pilocarpine-induced epilepsy model, the epileptiform burst firing in lateral septal and hippocampal neurons involves the activation of TRPC channels downstream from mGluRs, which is critical for the glutamate excitotoxicity (Phelan et al., 2012, 2013; Zheng and Phelan, 2014). In brain injury induced by focal cerebral ischemia, the roles of TRPC channels can be rather complex. For instance, while TRPC1 and TRPC4 mediate glutamate-induced neuronal death, TRPC6 may exert a protective role against ischemic neuronal death (Du et al., 2010).

Here, we explore the current evidence on contributions of TRPC channels in ischemic neuronal death, taking into account that these channels are activated downstream from mGluRs and likely work in concert with VGCCs and iGluRs to induce neuronal excitation and produce [Ca^2+^]_*i*_ signals, and such activities can have a pivotal impact on glutamate excitotoxicity. Because excitotoxicity occurs commonly in many types of neurodegenerative diseases, including Alzheimer’s disease, epilepsy, Huntington’s disease, and Parkinson’s disease, we also discuss the involvement of TRPC channels in these diseases in attempt to shed some lights on the mechanistic insights of TRPC regulation of excitotoxicity. Finally, we present some of our own data demonstrating the specific role of TRPC4/C5 channels in ischemic cell death and neuroprotective potential of targeting these channels using small molecular probes.

## The Basis of Excitotoxic Neuronal Death in Ischemic Stroke

Cerebral ischemic stroke is caused by interruption of blood supply through the middle cerebral artery (MCA). The MCA has large surface branches to supply blood into its territory that encompasses almost the entire boundary of the cortex and white matter of the hemisphere, including the major lobes (frontal, parietal, temporal, and occipital) and insula cortex (Brust, 2013). Occlusion of the MCA (MCAO) is the most frequently encountered stroke signs and symptoms, which may produce paralysis on the left or right side of the body, vision loss, and speech impairment (Rathore et al., 2002). The neurological deficits in ischemic stroke resulted from the deprivation of glucose and oxygen supply, or OGD, which leads to brain tissue damages. When the MCAO is prolonged, neurons lose glucose-dependent ATP generation, which in turn disrupts electrogenic pumps, e.g., plasma membrane Ca^2+^ ATPase (PMCA) and Na^+^/K^+^ ATPase.

The failure of the ATPases subsequently results in [Ca^2+^]_*i*_ increase and membrane depolarization, which causes glutamate release, as well as an elevation of extracellular K^+^ concentration. These also alter the ionic composition of the cytoplasm, with increases in Na^+^, Ca^2+^, and Cl^–^ and a decrease in K^+^ levels, leading to an osmolarity change and more commonly hyperosmolarity (Tanaka et al., 1997; Tanaka et al., 1999; Brisson and Andrew, 2012). Consequently, the inflow of water into the neuron in response to the osmolality change causes cell swelling and disruption of the membrane structure, which constitutes one of the multiple causes of neuronal death (Toyoda et al., 2020).

In addition to ionic imbalance caused by the failure of ATPases, excessive extracellular accumulation of glutamate also leads to toxic increases in [Ca^2+^]_*i*_, which activate multiple signaling pathways and ultimately lead to cell death (Durukan and Tatlisumak, 2007; Doyle et al., 2008). For many years, NMDA and AMPA receptors have been considered the pivotal targets of excitotoxicity associated with ischemic stroke. The activation of postsynaptic NMDA and AMPA receptors by synaptic glutamate causes membrane depolarization and Ca^2+^ influx, which definitely accounts for excitotoxicity. However, this is not the only way by which glutamate can elicit excitotoxicity. The abundant presence of mGluRs in both pre- and postsynaptic membranes, as well as extrasynaptic membranes, suggests that these metabotropic receptors are also activated under conditions of excessive glutamate release and accumulation both inside and outside the synapse. At least for mGluR1 and mGluR5, the activation will increase excitability through [Ca^2+^]_*i*_ elevation first by causing ER Ca^2+^ release. This will be followed by store- and/or receptor-operated Ca^2+^ entry, and in the case of coupling to TRPC channels, the activation of these mGluRs will also lead to membrane depolarization, given the non-selective nature of these channels, and the subsequent Ca^2+^ influx through both TRPC channels and VGCCs. Although permeable to both Na^+^ and Ca^2+^ as the NMDA and AMPA receptors, the conductance and activation kinetics of TRPC channels are vastly different from that of the iGluRs, typically being slower and longer lasting than the iGluRs as has been demonstrated during synaptic transmission (Hartmann et al., 2008; Riccio et al., 2009; Pressler and Regehr, 2013; Reiner and Levitz, 2018). Thus, in addition to iGluRs, the mGluR-TRPC coupling represents another main mechanism by which glutamate can induce excitotoxicity through [Ca^2+^]_*i*_ elevation and membrane depolarization, along with the consequent ionic imbalance, osmolarity changes and Ca^2+^ signaling (Toyoda et al., 2020), resulting in spatiotemporally distinct signaling effects that work in concert with those generated by iGluRs to induce cell demise.

**FIGURE 1:**
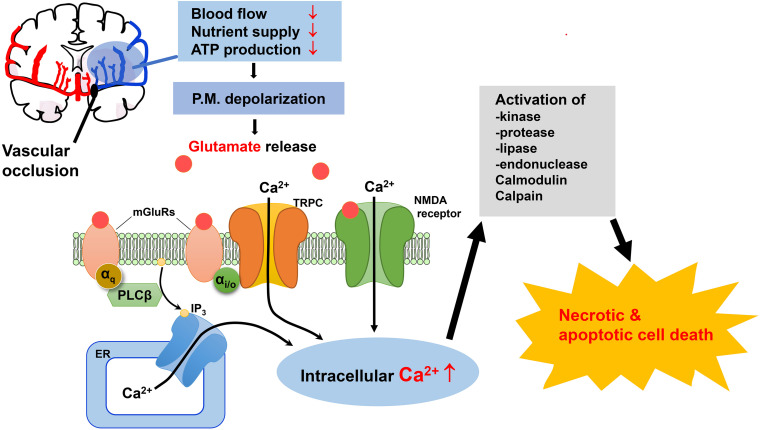
Possible mechanisms for the involvement of TRPC channels in excitotoxic neuronal death in ischemic stroke. Oxygen and glucose deprivation due to the blockade of blood flow to brain areas in ischemic stroke causes excess accumulation of extracellular glutamate. The subsequent activation of NMDA receptors and mGluRs causes elevation of intracellular Ca^2+^ levels through Ca^2+^ influx from extracellular space and Ca^2+^ release from the ER, respectively. TRPC channels are activated downstream from mGluRs, causing more sustained Ca^2+^ elevation by mediating additional Ca^2+^ influx. TRPC4 and TRPC5 channels are also activated downstream from mGluR subtypes that couple to PTX-sensitive G_*i/o*_ proteins, further expanding the repertoire of receptors that convey glutamate stimulation to Ca^2+^ signaling. Moreover, membrane depolarization resulting from activation of NMDA receptors and TRPC channels leads to excitation which also triggers additional Ca^2+^ influx through voltage-gated Ca^2+^ channels. The rise in intracellular Ca^2+^ due to these mechanisms causes activity changes of a plethora of enzymes, ion channels and transcription factors, as well as mitochondria, which when dysregulated induce cell death via either necrosis or apoptosis. ER, endoplasmic reticulum; IP_3_, inositol 1,4,5-trisphosphate; mGluRs, metabotropic glutamate receptors; NMDA, *N*-methyl-D-aspartate; P.M., plasma membrane; PTX, pertussis toxin.

Importantly, in spite of the promising results in reducing ischemic damage of brain tissues in animal models and the tremendous efforts made, antagonists of NMDA and AMPA receptors have not been effective in terms of neuroprotection against brain injury associated with ischemic stroke in clinical trials. Therefore, it would be beneficial to gain a better understanding of ischemic stroke pathology of the brain by exploring additional pathways, which will help identify new therapeutic candidates and approaches. Based on the current literature and our own experimental findings, we propose that TRPC channels play a critical part in neurological damage associated with ischemic stroke and should be evaluated as viable therapeutic targets for neuroprotection (Figure 1).

## The Roles of TRPC Channels in Cell Death

The seven mammalian TRPC members are divided into 4 subgroups (TRPC1, TRPC2, TRPC3/6/7, and TRPC4/5). Among them, TRPC1, C3, C4, C5, and C6 are highly expressed in neurons at various brain regions, with distinct distributions and functions; for example, TRPC3 is highly expressed in cerebellar Purkinje neurons and involved in motor coordination, TRPC4 and TRPC5 are highly expressed in cerebral cortex, hippocampus and amygdala and functionally implicated in anxiety and fear learning (Clapham, 2003; Venkatachalam and Montell, 2007; Hartmann et al., 2008; Riccio et al., 2009; Riccio et al., 2014). In general, the TRPC channels in neurons are activated downstream from stimulation of G_*q/*__11_-coupled receptors, including mGluR1/5 (Kim et al., 2003; El-Hassar et al., 2011; Phelan et al., 2012), M1 muscarinic receptors (Yan et al., 2009; Tai et al., 2011), 5-HT2 serotonin receptor (Munsch et al., 2003; Sohn et al., 2011; Gao et al., 2017), H1 histamine receptor (Tabarean, 2012), kisspeptin receptor (Zhang et al., 2008), cholecystokinin (CCK) type 1 and type 2 receptors (Riccio et al., 2009; Wang et al., 2011), and thyrotropin-releasing hormone (TRH) receptors (Zhang et al., 2015). In addition, receptor tyrosine kinases, such as TrkB receptor through stimulation by brain-derived neurotrophic factor (BDNF) (Amaral and Pozzo-Miller, 2007; Li et al., 2010) and leptin receptor by leptin (Qiu et al., 2010), are also linked to TRPC channel activation in neurons through activation of PLCγ’s. However, there are some exceptions. For instance, in olfactory bulb granule cells, the TRPC1/C4 heteromeric channels are dependent on NMDA receptors, instead of mGluRs, for activation that causes long lasting depolarization and sustained Ca^2+^ influx (Storch et al., 2012); the activation of TRPC-like channels in thalamic paraventricular nucleus neurons requires not only the G_*q/*__11_-coupled thyrotropin-releasing hormone receptors but also G_*i/o*_-coupled cannabinoid receptors CB1 and CB2 (Zhang et al., 2015). This latter observation is very interesting in light of the recent findings that receptor-operated activation of TRPC4-containing channels requires coincident stimulation of G_*i/o*_ protein signaling and PLC activities (Thakur et al., 2016, 2020; Jeon et al., 2020). In addition, some TRPC channels are also reported to be activated by nitric oxide, reactive oxygen species (ROS), thioredoxin, and Ca^2+^ store depletion (Yoshida et al., 2006; Xu et al., 2008; Ogawa et al., 2016). For more comprehensive discussions on activation mechanisms of TRPC channels, readers are referred to our recent review article (Wang et al., 2020).

All TRPC proteins form Ca^2+^-permeable non-selective cation channels, which are tetramers composed of either identical (homotetramers) or different (heterotetramers) TRPC subunits. Among the seven TRPC isoforms, TRPC1 has been the most frequently studied in the context of heteromeric channels (Storch et al., 2012), especially in the forms of TRPC1/C4 and TRPC1/C4/C5 heteromers (Phelan et al., 2012; Storch et al., 2012; Broker-Lai et al., 2017). However, TRPC1/C3 heteromers also form, at least in cortical astrocytes where they regulate astrogliosis in response to traumatic brain injury (Belkacemi et al., 2017). As is the case of TRPC1/C4 and TRPC1/C5 channels where TRPC1 reduced the channel conductance (Strubing et al., 2001), TRPC1 also serves to dampen the Ca^2+^ signal and the astrogliosis-promoting effect of TRPC3 (Belkacemi et al., 2017).

The activation of TRPC channels causes Na^+^ and Ca^2+^ influxes into the cell, leading to membrane depolarization and [Ca^2+^]_*i*_ elevation. Both effects are important for TRPC channels to carry out their physiological functions. However, under conditions when these effects were not properly controlled, they can also contribute to pathology, reminiscent of iGluRs and other channels that damage cells through disrupting the membrane potential and/or causing Ca^2+^ overload. Due to differences in the biophysical properties, mechanisms of regulation, cell-type specific expression and subcellular distributions, the amplitudes, durations and subcellular locations of the depolarization and Ca^2+^ signals brought about by the activation of TRPC channels are likely very different from that by other channels. Furthermore, besides membrane depolarization and Ca^2+^ signals, TRPC channels also exert their effects on cell survival through other mechanisms, such as direct physical interactions with proteins involved in the cell death pathways (see later). Thus, the TRPC channels can have unique contributions to cell damage in difference diseases and different experimental models.

At least for TRPC4, a self-propagating positive-feedback mechanism has been proposed that allows persistent and prolonged channel activation (Thakur et al., 2020). In lateral septal neurons, this results in typically a depolarization plateau reaching to about −5 mV that lasts for about 1 s even when the stimulus, an agonist of mGluR1/5, is given for only 30 ms (Tian et al., 2014a). The all-or-none feature of the depolarization plateau is also consistent with the positive feedback mechanism. It was shown that this mechanism involves complex interactions among several intracellular messengers and protein partners, e.g., Ca^2+^, H^+^, and PLCδ1 (Thakur et al., 2020), the specific ranges of Ca^2+^ and PIP_2_ concentrations as well as the optimal membrane potentials required for channel activation (Thakur et al., 2016). The functional coupling with PLCδ1 is probably a major reason for the self-propagating activation of the TRPC4 channel, as the PLC isozyme is targeted by its own products, Ca^2+^ and H^+^ (Thakur et al., 2020). PLC catalyzes the hydrolysis of PIP_2_, producing diacylglycerol (DAG), IP_3_ and H^+^ (Huang et al., 2010). Subsequently, IP_3_ releases Ca^2+^ from the ER store through activation of IP_3_ receptors and depletion of the ER Ca^2+^ store is often accompanied with store-operated Ca^2+^ entry from the extracellular space (Putney, 2017). When coupled to TRPC4, Ca^2+^ influx through the TRPC4 channel also provides an additional source for greater and more prolonged [Ca^2+^]_*i*_ increase, and the Ca^2+^ then feeds back to reinforce the activities of both PLCδ1 and TRPC4. In excitable cells, VGCCs also strongly impact native TRPC4 activation by bringing additional Ca^2+^, and perhaps also a part of the voltage sensitivity even though the TRPC channels are intrinsically voltage sensitive on their own (Gordienko and Zholos, 2004; Tian et al., 2014a). In addition, PIP_2_, which is continuously replenished upon hydrolysis through the actions of phosphatidylinositol 4 kinases and phosphatidylinositol-4-phosphate 5-kinases (Myeong et al., 2018), serves as both the membrane anchor and substrate of PLCδ1, as well as the source of DAG, IP_3_ and H^+^, which are all implicated in TRPC4 activation one way or the other (Storch et al., 2017; Thakur et al., 2020). However, at high concentrations, PIP_2_ also exerts a tonic block on TRPC4 activation (Otsuguro et al., 2008; Thakur et al., 2016), and similarly, high cytosolic Ca^2+^ concentrations also suppress the channel activity (Thakur et al., 2016, 2020). Moreover, the optimal membrane potentials for TRPC4 activation, especially native TRPC4-containing channels, have been observed at −40 to −60 mV, and are influenced by [Ca^2+^]_*i*_ (Gordienko and Zholos, 2004). Therefore, an intricate balance is created through complex interactions among Ca^2+^, PIP_2_, DAG, H^+^, and membrane voltage to support the coupled activities of PLCδ1 and TRPC4, leading to sustained channel activity that lasts much longer than the time of exposure to the triggering stimuli. Notably, for TRPC4, the triggering stimuli include coincident activations of G_*i/o*_ proteins and PLC signaling through either G_*q/*__11_-PLCβ or receptor tyrosine kinase-PLCγ pathways (Jeon et al., 2020). For other TRPC channels, G_*i/o*_ proteins may not be involved and the depolarization amplitude and duration may not be as strong and as long, respectively, as that of TRPC4. However, the dual (both stimulatory and inhibitory) regulation by PIP_2_ and Ca^2+^, and the sensitivity to DAG are common among the TRPCs (Wang et al., 2020), although whether or not they exhibit a specific dependence on a specific PLC isozyme, as in the case of PLCδ1 for TRPC4 (Thakur et al., 2016), remains to be elucidated. It is possible that other TRPC channels also exhibit self-propagating activation like TRPC4, but with different kinetics, amplitudes, and durations.

At the cellular level, the most direct evidence of TRPC channel contribution to cell death comes from cytotoxic effect of (−)-englerin A, a guaiane sesquiterpenoid found in the bark of *Phyllanthus engleri*, on renal carcinoma cells (Akbulut et al., 2015; Carson et al., 2015) and triple-negative breast cancer (TNBC) cell lines (Grant et al., 2019) that express high levels of TRPC4 and/or TRPC1. It was found that englerin A exerts its cytotoxic effect through activation of TRPC4 and TRPC1/C4 channels endogenously expressed in the cancer cells. Intuitively, the cell killing action of englerin A was attributed to TRPC4-mediated Ca^2+^ influx and then intracellular Ca^2+^ overload, as Ca^2+^ overload by TRPC channels has indeed been reported to regulate apoptosis or other forms of programmed cell death in some cell types (Kondratskyi et al., 2015; Maher et al., 2018; Elzamzamy et al., 2020). However, a later study suggested that the cytotoxicity effect might largely result from the excessive Na^+^ influx (Ludlow et al., 2017). It would appear that both the Na^+^ and Ca^2+^ influxes mediated by TRPC channels are potentially harmful to the cell, if not properly controlled.

## The Role of TRPCs in Neurodegenerative Diseases Aside From Stroke

Pathologically, excess glutamate accumulation is relevant to brain injury in many forms of neurodegenerative diseases. Therefore, before discussing the possible contributions of TRPC channels to brain injury associated with ischemic stroke, it is worthwhile to consider the involvement of TRPC channels in neuronal cell death in other neurodegenerative diseases, as they likely share some common mechanisms of excitotoxicity with ischemic stroke in damaging brain neurons. TRPC channels have been reported to play roles in a number of neurological disorders. Depending on the type of disease and the specific TRPC subtype, the activation of TRPC channels is not always detrimental to the neuron. Some TRPCs may actually be neuroprotective in certain neurodegenerative diseases.

A likely consequence of pathological accumulation of excess glutamate is coincident stimulation of both G_*q/*__11_- and G_*i/o*_-coupled mGluRs. This provides conditions for strong and prolonged self-propagating activation of TRPC4 and TRPC5 channels, leading to membrane potential disruption and Ca^2+^ overload. Indeed, TRPC1/C4 and TRPC5 channels have been implicated in seizure-induced neuronal death in mouse lateral septum and hippocampus, respectively, in the pilocarpine-induced model of epilepsy (Phelan et al., 2012). Inhibiting TRPC5 also protected neurons in pyriform cortex, amygdala, and hippocampus from death in kainate-treated rats with prolonged seizures (Park et al., 2019) and in hippocampal CA3 neurons in a tramatic brain injury model (Park et al., 2020). In these models, TRPC5 was thought to be activated by oxidation which first triggered a rise in cytosolic Zn^2+^ levels and then opening of TRPC5 channels to mediate Ca^2+^ influx, leading to neuronal cell death (Park et al., 2019). In the Huntington’s disease model, activation of TRPC5 through glutathionylation has also been shown to cause the loss of striatal neurons (Hong et al., 2015), and this effect can be mitigated through destabilizing the presence of TRPC5 on plasma membrane via depalmitoylation (Hong et al., 2020).

On the other hand, TRPC4-containing channels are not always associated with neuronal cell injury. TRPC1/C4 heteromeric channels have been reported to exert a protective role in neuronal cell death induced by subarachnoid hemorrhage (Wang et al., 2016). This was suggested to occur through Ca^2+^ activation of calcineurin which suppresses NMDA receptor activity through dephosphorylation. Likewise, TRPC1 has been found to be neuroprotective in animal models of Parkinson’s disease and Alzheimer’s disease. In dopaminergic neurons of substantia nigra, TRPC1 inhibits L-type VGCCs by facilitating the interaction of STIM1 with Cav1.3 in response to ER Ca^2+^ store depletion and thereby dampening the neurotoxin-induced VGCC function that causes neuronal death (Sun et al., 2017). In addition, through store-operated Ca^2+^ entry, TRPC1 protects the dopaminergic neurons from neurotoxin-induced ER stress and the decrease of AKT/mTOR signaling (Selvaraj et al., 2012). Conversely, dopaminergic neurons respond to neurotoxin with decreased store-operated Ca^2+^ entry via attenuation of TRPC1 transcription, which is regulated by NF-κB (Sukumaran et al., 2018). Furthermore, the inhibition of TRPC1-mediated Ca^2+^ entry by neurotoxin is facilitated by sigma 1 receptor, an ER membrane protein acting as a chaperone to regulate Ca^2+^ release and other functions, which ultimately leads to the loss of dopaminergic neurons (Sun et al., 2020). In the mouse model of Alzheimer’s disease, the deletion of *trpc1* gene was found to aggravate amyloid-β (Aβ)-induced learning and memory deficits and TRPC1 was shown to interact with Aβ precursor protein (APP) at the transmembrane region, resulting in reduced Aβ levels in hippocampal neurons and attenuation of apoptosis (Li et al., 2018). By contrast, the upregulated TRPC1 expression seen in SK-N-SH human neuroblastoma cells expressing a polyglutamine Huntingtin mutant was found to support a store-operated non-selective cation current and this activity contributed to glutamate-induced apoptosis of primary cultured striatal medium spiny neurons prepared from YAC128 mice that mimic neurodegeneration of Huntington’s disease (Wu et al., 2011). Indeed, eliminating TRPC1 in YAC128 mice improved motor performance and rescued neuronal spines from progressive loss (Wu et al., 2018). Therefore, TRPC1 may be either detrimental or beneficial to brain neurons depending on the models and neuronal cell types.

TRPC3 has been speculated to play a part in ischemic injury of the cerebellum owning to it higher expression in this than in other brain regions (Cederholm et al., 2019). However, this regional specific effect of TRPC3 was found to change in aging monkeys and transgenic mice overexpressing human α-synuclein, where TRPC3 was found to become enriched in the mitochondria of striatal neurons and contribute to the disruption of mitochondrial membrane potential and cell apoptosis commonly seen in Parkinson’s disease (Chen M. et al., 2017). Thus far, TRPC3 represents the only TRPC isoform that has been localized to mitochondria, in addition to its common plasma membrane localization, and involved in Ca^2+^ uptake into the organelle (Feng et al., 2013). Thus, the link of TRPC3 expression and function in mitochondria to α-synuclein upregulation implicates a role of TRPC3 in the pathogenesis of Parkinson’s disease (Chen M. et al., 2017). In a rodent model of pilocarpine-induced status epilepticus, while the expression of TRPC3 was found to be increased, that of TRPC6 was decreased in CA1 and CA3 pyramidal neurons and dentate granule cells. It was reported that either inhibiting TRPC3 or enhancing TRPC6 function and/or expression protected neurons from seizure-induced injury (Kim et al., 2013). These findings support the detrimental effect of TRPC3 hyperactivation on brain neurons, but implicate that TRPC6 may exert an opposite effect.

Indeed, although closely related to TRPC3, TRPC6 has mainly been shown to be protective against neurodegeneration. For example, a system wide reduction of TRPC6 expression was found in patients with Alzheimer’s disease and mild cognitive impairment, including blood cells, which negatively correlated with the cognitive performance (Lu et al., 2018; Chen et al., 2019). Moreover, neurons differentiated from induced pluripotent stem cells (iPSCs) derived from peripheral blood of sporadic Alzheimer’s disease patients also exhibit decreased TRPC6 expression, as well as elevated Aβ and phosphorylated tau levels, hallmarks of Alzheimer’s disease (Tao et al., 2020). Mechanistically, TRPC6 has been proposed to physically interact with APP via its second transmembrane segment, through which it inhibits the cleavage of APP by γ-secretase, leading to reduced Aβ production (Wang et al., 2015). That increasing TRPC6 expression or using a membrane penetrating peptide representing TRPC6’s second transmembrane segment effectively lowered the levels of Aβ and phosphorylated tau in iPSC neurons derived from sporadic Alzheimer’s disease patients supports a role of TRPC6 in suppressing the disease pathogenesis (Tao et al., 2020).

In a separate study, TRPC6 was found to form a complex with Orai2 that is regulated by STIM2 to conduct store-operated Ca^2+^ influx in dendritic mushroom spines of hippocampal neurons (Zhang et al., 2016). When this is impaired, the mushroom spines become unstable and disrupted, leading to memory loss. The beneficial effect of TRPC6 to Alzheimer’s disease was demonstrated by showing that stimulating TRPC6 or the store-operated Ca^2+^ entry improved hippocampal long-term potentiation of the APP-presenilin 1 mutant mice, an experimental model of Alzheimer’s disease (Zhang et al., 2016; Popugaeva et al., 2019). However, in a different model, where the expression of a familial Alzheimer’s disease (FAD) presenilin 1 mutation bearing the deletion of the 9th exon (PSEN1ΔE9) in hippocampal neurons caused mushroom spine loss through enhancing, instead of suppressing, TRPC6-mediated store-operated Ca^2+^ entry, inhibiting such activity then becomes beneficial (Chernyuk et al., 2019). Therefore, depending on the nature of dysregulation, TRPC6 can be either beneficial or detrimental to neurons, highlighting the importance of maintaining the proper Ca^2+^ homeostasis. Previously, an inhibitory effect of presenilin 2 and its Alzheimer’s-disease-linked variants on receptor-operated TRPC6 function has been demonstrated in heterologous expression systems, suggesting functional interaction between presenilins and TRPC6 (Lessard et al., 2005). In status epilepticus, TRPC6 serves to protect granule neurons of dentate gyrus from degeneration through activation of ERK1/2 and the subsequent phosphorylation of dynamin-related proteins 1 (DRP1) at Ser-616 (Ko and Kang, 2017) and an increase in the expression of a mitochondrial protease, Lon protease-1 (Kim et al., 2019). In the absence of TRPC6, the hypo-phosphorylation of DRP1 and reduction in Lon protease-1 then lead to mitochondrial elongation and dysfunction, which increase the vulnerability of granule cells to seizure-induced death (Kim and Kang, 2015).

Taken together, the above findings suggest that TRPC channels exert differential effects on neurodegeneration in isoform and disease specific fashions. Although [Ca^2+^]_*i*_ dysregulation and membrane depolarization may be the common effects of TRPC channel activation, other mechanisms, including protein–protein interactions that involve specific regions of particular TRPC subtypes with certain disease-related protein partners and unique signaling pathways pertaining to the regulation of specific TRPC channels, are also involved. These diverse mechanisms underlie the differential outcomes resulting from TRPC channel function, ranging from neuroprotection to neurodegeneration. Therefore, it cannot be generalized that the extended activation of TRPC channels always leads to cell death, despite the high risk of Ca^2+^ overload.

## The Role of TRPCs in Ischemic Brain Injury

Brain injury after focal ischemic insult has been linked to the reduction of oxygen and nutrient (glucose) supply to the affected brain region (Lipton, 1999; Lo et al., 2003). Both *in vivo* MCAO model and the *in vitro* OGD model are commonly used to evaluate the involvement and mechanisms of TRPC channels in neuronal cell death associated with ischemic stroke. Like with the other neurodegenerative diseases discussed above, these studies revealed complex roles of different TRPC channels in ischemic brain injury.

For TRPC1, both the MCAO model and OGD assay suggested that TRPC1 plays a protective role against neuronal injuries caused by cerebral ischemia/reperfusion through suppression of ROS generation (Xu et al., 2018). TRPC1 expression was shown to be downregulated not only in brain tissues of mice subject to 90 min MCAO followed by 24 h reperfusion, but also in the cultured murine hippocampal cell line, HT22, that was exposed to an OGD culture for 4 h and then placed in the reoxygenated normal medium for 6–24 h. It was demonstrated that the amplitude of store-operated Ca^2+^ entry in the hippocampal cells was positively corrected with the expression TRPC1, but negatively correlated with the NADPH oxidase activity, and unrelated to the expression of STIM1 and Orai1 as well as mitochondrial ROS generation. However, no evidence was provided to suggest a direct inhibitory effect of Ca^2+^ on NADPH oxidase activity. Rather, TRPC1 inhibited NADPH oxidase-mediated ROS production through physically interacting with a catalytic component of the oxidase, Nox4, which facilitated Nox4 degradation along with cytoplasmic retention of the cytosolic subunits of the NADPH oxidase complex, p47phox and p67phox (Xu et al., 2018). This protective role of TRPC1 is opposite from the results of an earlier work, which showed that SKF96365 prevented cortical neuron death induced by 1.5 h OGD and 24 h reoxygenation (Wang et al., 2016). Since SKF96365 is a non-specific drug that inhibits all TRPC channels, and all forms of store- and receptor-operated Ca^2+^ entry, it did not inform which specific channel type was responsible for the protective effect.

On the other hand, the TRPC3/6/7 triple knockout mice were reported to be resistant to brain injury induced by MCAO followed by reperfusion (Chen X. et al., 2017). Here, the detrimental role of TRPC3/6/7 channels was attributed at least in part to astrocytes, in which these channels contributed to the enhanced NF-κB phosphorylation, reduced AKT phosphorylation and increased cell apoptosis following OGD and reoxygenation treatment (Chen X. et al., 2017). How this function of TRPC3/6/7, which promotes the death of astrocytes, is related or coordinated with astrogliosis, an abnormal proliferation of brain astrocytes observed after stroke and shown to be positively regulated by TRPC3 (Shirakawa et al., 2010; Munakata et al., 2013; Belkacemi et al., 2017), is an interesting question that needs to be addressed in future studies.

Although TRPC6 expression in mouse cortical neurons has been reported to be increased following brain ischemia/reperfusion *in vivo* and OGD/reoxygenation *in vitro*, and this increase contributed to neuronal injury in an NMDA receptor-dependent manner (Chen J. et al., 2017), nearly all other studies argue for a protective role of TRPC6 in neurological damage associated with ischemic stroke. The neuroprotective role of TRPC6 was suggested to occur through activation of cAMP response element-binding protein (CREB) signaling, which is disrupted following cerebral ischemia due to degradation of TRPC6 proteins in neurons by proteolytic cleavage mediated by calpain, non-lysosomal cysteine proteases activated as a result of [Ca^2+^]_*i*_ rise from NMDA receptor activation by elevated extracellular glutamate (Du et al., 2010). It was found that attenuating the calpain-mediated TRPC6 degradation could underlie the neuroprotective effects of several natural compounds, such as resveratrol, neuroprotectin D1, and (–)-epigallocatechin-3-gallate (the main ingredient of green tea), and calycosin (Lin et al., 2013a; Yao et al., 2013, 2014; Guo et al., 2017). That these protective effects were obliviated by inhibiting mitogen-activated protein kinase kinase (MEK) and/or calmodulin kinases suggests that these kinases mediate CREB activation downstream from TRPC6 (Lin et al., 2013b; Yao et al., 2013). On the other hand, increasing TRPC6 expression or its function through overexpression or treatment with hyperforin also inhibited NMDA receptor function and suppressed calpain activity (Li et al., 2012; Lin et al., 2013b), indicative of reciprocal regulations between TRPC6 and NMDA receptors/calpain. Once suggested as a TRPC6 agonist (Leuner et al., 2007), hyperforin was found to increase TRPC6 expression in mouse hippocampus following ischemia/reperfusion (Lin et al., 2013b). Hyperforin was also reported not to change TRPC6 channel activity, but acts independently of TRPC6 as a protonophore, which may explain its anti-depressive effects (Sell et al., 2014). Furthermore, the calpain cleavage of TRPC6 has also been reported to be activated by interleukin 17 (IL-17) (Zhang et al., 2014), a T-cell derived pro-inflammatory cytokine shown to contribute to ischemic brain injury (Gelderblom et al., 2012), suggesting an immune-neural interaction that exacerbates ischemic neurological damage through downregulating TRPC6.

In contrast to the rich information on TRPC6, little is known about the roles of TRPC4 and TRPC5 in brain injury associated with ischemic stroke. In one study, it was reported that TRPC4 protein expression was increased in rat striatal and hippocampal neurons at 12 h to 3 days after MCAO (Gao et al., 2004). Other studies speculating a detrimental function of TRPC5 in neurological deficits following stroke had based on the activation of TRPC5 by oxidation, without experimentation in the stroke model (Ishii et al., 2011; Park et al., 2019, 2020). In a very recent study, TRPC5 was found to directly interact with phospholipid scramblase 1 (PLSCR1) on the plasma membrane to facilitate externalization of phosphatidylserine (PS) and apoptosis of cortical neurons in response to cerebral ischemia/reperfusion (Guo et al., 2020). The same protein complex likely also includes TRPC1 and TRPC4. While the examination of cerebral slices of animals subject to ischemia/reperfusion revealed reduced PS externalization and apoptosis in TRPC5 knockout mice, whether these translate into protection against cerebral infarction and/or behavioral neurological impairments remains to be elucidated (Guo et al., 2020). On the other hand, overexpression of TRPC5 in spinal cord via adeno-associated viruses attenuated spinal cord ischemia/reperfusion injury in rats (Shen et al., 2020). This could result from the angiogenic function of TRPC5 which dampens the injury induced inflammation. Indeed, TRPC5 has been reported to promote endothelial cell sprouting, angiogenesis, and blood perfusion in ischemic tissues through activation of nuclear factor of activated T cell (NFAT) isoform c3 and angiopoietin-1 (Zhu et al., 2019). Therefore, the effect of TRPC4/C5 on ischemic brain injury may be very complex, including both positive and negative regulations that either exacerbate or prevent neuronal cell death.

## TRPC4 in Ischemic Brain Injury – Our Own Study

The above analysis indicates that despite the common mechanisms that link G protein and PLC pathways to TRPC channel activation and the likelihood that these channels participate in excitotoxicity triggered by excessive extracellular accumulation of glutamate through mGluRs, it is not easy to predict whether a specific TRPC channel type is detrimental or beneficial to brain neurons under conditions of cerebral ischemia/reperfusion. Depending on the TRPC isoforms, cell types involved, and mechanisms of actions, the TRPC channel may be neuroprotective or destructive.

**FIGURE 2:**
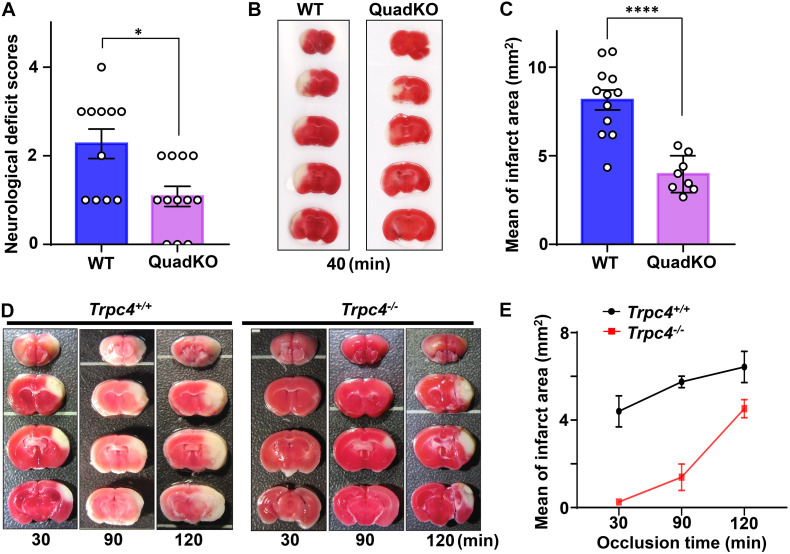
Transient receptor potential canonical (TRPC) deficiency attenuates cerebral ischemic damage. **(A–C)** TRPC1, C4, C5, C6 quadruple knockout (QuadKO) and wild type control mice were subjected to reversible MCAO for 40 min using intraluminal suture method, followed by 22–24 h reperfusion. After assessing neurological deficits as described (Li et al., 2011) **(A)**, the animals were sacrificed and brain sections stained with TTC for quantification of infarct areas **(B,C)**. **(A)** Neurological scores of animals subjected to ischemia/reperfusion. Data represent means ± SEM for *n* = 11 (wild type) and 12 (QuadKO) mice. ^∗^*P* < 0.05 by Mann–Whitney test. **(B)** Representative photographs of TTC-stained brain sections demarcating infarction. **(C)** Infarct areas at rostrocaudal plains were determined from morphometric analyses of TTC-stained brain sections. Data represent means ± SEM for *n* = 12 (wild type) and 8 (QuadKO) mice. ^****^P < 0.0001 by unpaired *t*-test. **(D,E)** *Trpc4*^–/–^ and wild type control mice were subjected to reversible 30, 90, and 120 min MCA/CCAO, followed by 24 h reperfusion. **(D)** Representative photographs of TTC-stained brain sections. **(E)** Infarct areas at rostrocaudal plains were determined from morphometric analyses of TTC-stained brain sections. Data are presented as means ± range for *n* = 2 mice for each time point.

To define the overall contributions of TRPC channels in brain damage associated with cerebral ischemia, we first compared neurological scores and brain infarction volumes of wild type and quadruple TRPC1, C4, C5, C6 knockout (QuadKO) mice subject to 40 min of MCAO using the standard intraluminal suture (thread) method, followed by 24 h reperfusion. In this method, a monofilament nylon surgical suture with a heat-rounded tip is inserted from an opening at external carotid artery and advanced through internal carotid artery until it reaches the middle cerebral artery to block the blood flow there. After the desired time period of occlusion, the filament was withdrawn to allow resupply of the blood to the blocked brain area (Alkayed et al., 1998; Sun et al., 2019). Since TRPC2 is mainly expressed in the vomeronasal system (Liman et al., 1999), TRPC3 mainly present in the cerebellum (Hartmann et al., 2008), and TRPC7 mostly found in peripheral tissues (Okada et al., 1999), we consider TRPC1, C4, C5, and C6 as the main cerebral TRPC subtypes expressed in areas typically affected in the MCAO model. As described above, both TRPC1 and C6 have been suggested to play protective roles against ischemic brain injury, but the function of TRPC4 and C5 remains unclear. Therefore, the QuadKO mice provide a good model to learn if TRPC channels generally serve a protective or detrimental role in cerebral ischemia. We found that on average, TRPC-QuadKO mice had better neurological scores (Figure 2A) and smaller brain infarct areas (shown by staining with 2,3,5-triphenyltetrazolium chloride, TTC) than the wild type mice (Figures 2B,C), suggesting that overall, TRPC channels contribute to causing damage of the brain in response to cerebral ischemia/reperfusion. Given that TRPC1 and TRPC6 may play some neuroprotective function during ischemia/reperfusion, it is plausible that the detrimental actions are mainly mediated by TRPC4 and/or TRPC5, and these two TRPC isoforms may dominate the cortical neuronal responses to ischemic insults. This interpretation is consistent with the finding that TRPC4/C5 expression is more abundant than other TRPC isoforms in several major areas of rat brain, including the prefrontal cortex (Fowler et al., 2007). Clearly, the distinct contributions of different TRPC isoforms in brain injury at different stages of cerebral ischemia/reperfusion warrant more detailed investigation.

Given that among the major brain TRPC isoforms, only TRPC4 has not been evaluated for its role in ischemic stroke, we then set out to compare brain damages caused by cerebral ischemia/reperfusion in wild type and TRPC4 knockout mice. Here, we performed transient focal middle cerebral artery/common carotid artery occlusion (MCA/CCAO) on the animals for 30, 90, and 120 min, followed by 24 h reperfusion. The MCA/CCAO is surgically performed using craniotomy to directly occlude distal MCA with a thin stainless wire (Aronowski et al., 1994; Zhao et al., 2015). This type of method was reported to produce reversible infarction and confined focal cerebral ischemia at distal MCA territory (Braeuninger and Kleinschnitz, 2009; Tavafoghi et al., 2016). We found that the infarct areas were markedly smaller in brains of *Trpc4*^–/–^ mice than wild type animals, especially with shorter time of ischemia, i.e., 30 and 90 min (Figures 2D,E). Specifically, with the 30 min ischemia followed by 24 h reperfusion, whereas the infarctions were readily visible in wild type brains, they were hardly detectable in *Trpc4*^–/–^ specimens. However, with a longer time (120 min) ischemia, the protective effect of TRPC4 knockout diminished (Figures 2D,E). These data indicate that TRPC4-containing channels contribute positively to ischemic brain damage, particularly during the early time period of the ischemia.

**FIGURE 3:**
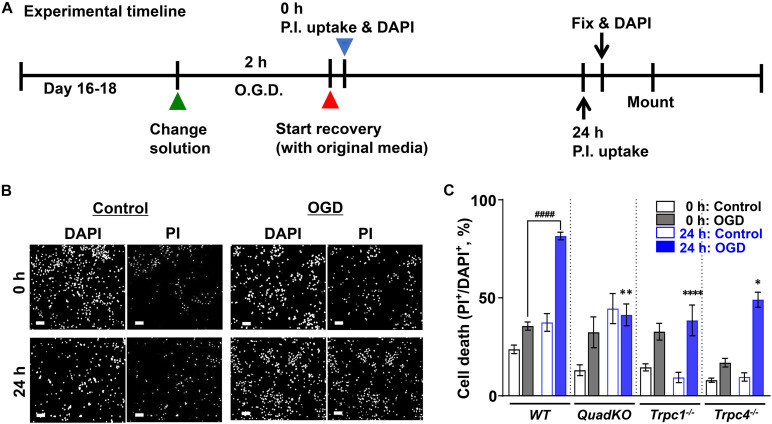
Oxygen and glucose deprivation (OGD)-induced cell death of cultured cortical neurons from wild type, *Trpc1*^–/–^, *Trpc4*^–/–^, and *Trpc1/c4/c5/c6*^–/–^ mice. **(A)** Schematic of the experimental timeline. Primary cultures of cortical neurons (16–18 DIV) from WT, *Trpc1*^–/–^, *Trpc4*^–/–^, and *Trpc1/c4/c5/c6*^–/–^ (QuadKO) mice were treated without or with OGD for 2 h and then allowed to grow in normal medium for 0 or 24 h. **(B)** Representative images of DAPI staining (Bocksch et al., 2010) and PI uptake of cultured cortical neurons from wild type (WT) mice grown under control conditions or subjected to OGD for 2 h. Cells were stained either immediately after the treatment (0 h) or returned to normal culture conditions for 24 h before staining (24 h). Scale bars, 50 μm. **(C)** Summary data of PI-positive cells, normalized to the total cell number determined by DAPI staining. Data represent means ± SEM of *n* = 20 to 30 fields of views for each condition pooled from 4 coverslips of neurons from two separately prepared primary cultures. ^####^*P* < 0.0001, ^∗^*P* < 0.05, ^∗∗^*P* < 0.01, ^****^P < 0.0001 vs. WT 24 h: OGD by one-way ANOVA.

Next and to attribute underlying mechanisms more directly to TRPC4 function in neurons, we used cortical neuron cultures prepared from newborn wild type, *Trpc1*^–/–^, *Trpc4*^–/–^ and TRPC QuadKO mice to examine the effect of OGD-reoxygenation on cultured neurons *in vitro* (Ying et al., 1997). As illustrated in Figure 3A, cells were cultured for 16–18 days *in vitro* (DIV). On the day of the experiment, the culture medium was replaced with either a deoxygenated bicarbonate buffer without glucose (OGD) or an oxygenated bicarbonate buffer with glucose (Control), and the cultures were maintained in a 37°C hypoxic incubator for 2 h with the oxygen level kept at 1% atmosphere. After the treatment, the neurons were either immediately evaluated for cell death by staining with propidium iodide (PI) or returned to the normal culture medium and maintained in the regular incubator at the normal oxygen level for 24 h before the PI staining. All cells were also stained with DAPI (4′,6-diamidino-2-phenylindole) to label the nuclei (Figure 3B). A comparison of the ratios of PI-positive/DAPI-positive labels, which represent percental cell death, revealed that wild type neurons were more vulnerable than the TRPC knockout neurons to the 2 h exposure of the bicarbonate buffer no matter if the buffer was oxygenated or not (Figure 3C). The reason for this is unclear, but could be related to the differences in the developmental status between the wild type and TRPC knockout cortical neurons cultured *in vitro*. Indeed, TRPCs play roles in several different aspects of neuronal development (Greka et al., 2003; Jeon et al., 2013; Tian et al., 2014b) and some of these could influence the sensitivity of the neurons to nutrient deprivation, given that the bicarbonate buffer does not contain serum and amino acids. This protective effect of TRPC depletion on neuronal survival in the serum and amino acid-deprived but glucose-containing buffer requires further investigation. Despite this, while the 2-h OGD treatment (without reoxygenation) did not immediately increase cell death further in wild type cortical neurons, it did so in all TRPC knockout neurons (Figure 3C), suggesting that OGD may interfere with the protective effect of TRPC knockout on cultured cortical neurons subject to serum and amino acid deprivation.

With the 24 h reoxygenation and nutrient replenishment (herein referred to as refeeding), cell death nearly doubled in wild type neurons that had been treated with OGD but not those exposed to the control buffer. The response to refeeding, however, was quite different between the three different TRPC knockout lines. The control buffer-treat QuadKO neurons displayed a marked increase in cell death after 24 h refeeding, but OGD-treated ones virtually did not change. However, while the *Trpc1*^–/–^ neurons did not show any increase in death after refeeding regardless of the oxygen and glucose status during the starvation, the *Trpc4*^–/–^ neurons exhibited a marked refeeding-dependent increase in cell death only when OGD treatment was applied (Figure 3C). These results, although difficult to interpret without more extensive investigations, underscore the complex and diverse roles different TRPC channels play in neuronal cell responses to nutrient shortage, OGD, and the subsequent replenishment of all nutrients, including oxygen.

**FIGURE 4:**
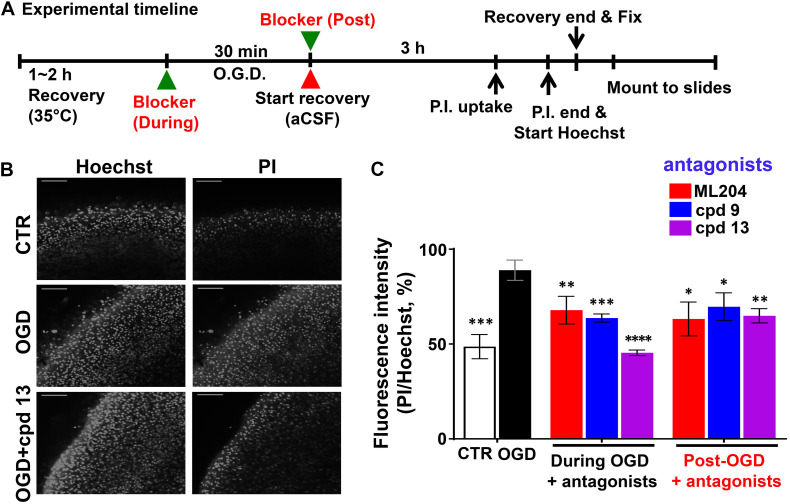
Oxygen and glucose deprivation -induced neuronal death in the cerebral cortex of mouse brain slices. TRPC4/C5 blockers rescued OGD-induced neuronal death in the cerebral cortex of brain slices. **(A)** Schematic of the experimental timeline. **(B)** Representative images of Hoechst staining (left panels) and PI uptake (right panels) of acute brain slices (P15) from wild type (WT) mice without (CTR) or with OGD for 30 min in the absence or presence of TRPC4/C5 blocker, compound 13 (cpd 13). After the treatment, the slices were recovered for 3 h and stained with PI and Hoechst at 40 and 10 min, respectively, before the end of the 3 hr incubation. Scale bars, 100 μm. **(C)** Summary data of PI/Hoechst fluorescence ratios (random unit, but the same settings used for all conditions). TRPC4/C5 blockers, ML204 (at 50 μM), cpd 9, and cpd 13 (at 30 μM) prevented the OGD-induced cell death to varying degrees no matter if they were added during or after (post) the OGD treatment. Data represent means ± SEM of *n* = 8 to 15 fields of view of independently treated slices. ^∗^*P* < 0.05, ^∗∗^*P* < 0.01, ^∗∗∗^*P* < 0.001, ^****^P < 0.0001 vs. OGD without antagonist by Student *t*-test.

To avoid the complications with the cultured neurons and the developmental effects of the TRPC knockout, we also performed OGD experiments using brain slices following a previous protocol (Taylor et al., 1999; Tasca et al., 2015). As illustrated in Figure 4A, the cerebral slices from wild type mice were recovered in artificial cerebrospinal fluid (aCSF) bubbled with 95% O_2_ and 5% CO_2_ at 35°C for 1–2 h after cutting. Then they were transferred to a modified aCSF omitting glucose and bubbled with N_2_ (OGD) and maintained for 30 min at the same temperature. Control slices were treated with fresh oxygenated aCSF in parallel. After that, all slices were returned to fresh oxygenated aCSF and incubated for another 3 h. PI and Hoechst were added at 40 and 10 min, respectively, before the end of the incubation to label dead cells and nuclei of all cells. This was followed by fixation with 4% paraformaldehyde (12 h at 4°C) and mounting. In order to examine to what extent inhibiting TRPC4/C5 channels can protect against OGD-induced neuronal death and the possible therapeutic windows of targeting these channels, we also applied TRPC4/C5 antagonists, ML204 (Miller et al., 2011) and compounds 9 and 13 (cpd 9, cpd 13, analogs of M084) (Zhu et al., 2015), either at the beginning or the end of the OGD treatment. As shown in Figures 4B,C, after 30 min OGD and 3 h reoxygenation, the number and intensity of PI labeling in the cortex area were dramatically increased as compared to the control. Including the TRPC4/C5 antagonists either during or post OGD significantly decreased the damage. Since the TRPC4/C5 antagonists inhibit both TRPC4 or TRPC5 homomeric channels and TRPC1/C4 and TRPC1/C5 heteromeric channels, these data support the general idea that targeting TRPC1/C4/C5 channels can be beneficial to stroke therapy including post ischemic protection. Clearly, additional studies are needed to illustrate the details on how these TRPC channels contribute to neurological damage in response to ischemia and reperfusion and the appropriate intervention strategies that bring the most benefit with minimal detrimental effects.

## Discussion

Recent studies have demonstrated that TRPC channels play complex and even opposing functions in neuronal cell survival and death. Depending on the different TRPC subtypes, brain locations and cell types, as well as the specific pathological conditions, TRPC channels can be either detrimental or beneficial to neurons. However, the general theme is that for nearly every neurodegenerative disease, TRPC channels play crucial roles, and these include ischemic stroke and other conditions that cause brain injury. Because of the diverse mechanisms that have been revealed so far, it is also clear that no generalized mechanism should be used to explain or predict how a specific TRPC channel contributes to brain injury. Even for TRPC6, although most studies have indicated a pro-survival role, the molecular and cellular mechanisms elucidated from various studies have been very different, ranging from a direct interaction of the channel with a specific disease-causing protein (Wang et al., 2015), regulation of mitochondrial fission (Ko and Kang, 2017), to CREB-dependent transcription (Du et al., 2010) and suppression of NMDA receptor and calpain function (Li et al., 2012; Lin et al., 2013b). Therefore, future studies should focus not only on unique mechanism(s) that underlies the regulation of a specific TRPC channel in a particular form of neuronal injury, but also make attempts to reconcile the mechanism with existing knowledge and identify a common theme(s), if possible, that would enrich the mechanistic understanding of how the TRPC channel contributes to disease pathogenesis.

Particularly for brain damage associated with ischemic stroke, the roles of TRPC channels at different stages of the ischemia/reperfusion and in different brain areas are far from clear. The acute deprivation of oxygen and glucose, as well as other nutrient supplies, certainly exerts a tremendous stress on neurons, causing many changes in signal transduction and intracellular and extracellular messengers that are linked to TRPC channels through either functional or physical coupling or both. These can be factors that directly activate the channel, such as DAG or ROS, or transmitters that work through G protein-coupled receptors, like glutamate, as well as neurotrophic factors that stimulate tyrosine kinases, e.g., BDNF. The combination of some of these factors may even bring synergistic effect, making some otherwise insignificant molecules into important contributors to TRPC channel activation that either induces neuronal death or protects them from the damages caused by other signals. Importantly, changes that occur in the ischemic stage do not just revert back to their original states after reperfusion or oxygenation. The restoration of nutrient supply and blood circulation also causes inflammation and oxidation, leading to stress responses that may evoke TRPC activation as well. Thus, a TRPC subtype may be involved in either the ischemic or reperfusion stage, or both stages, of the ischemia/reperfusion injury but with different outcomes. Furthermore, while irreversible damages can occur immediately at the ischemic core during OGD through necrosis, neurons in the neighboring regions (ischemic penumbra) more often display delayed cell death or apoptosis (Rami and Kogel, 2008). It is not known how different TRPC isoforms are involved in the different forms of cell death pathways, and therefore, it remains to be elucidated whether and how each of them plays a part in different stages and different brain regions (core *vs*. penumbra) in neurological damages associated with ischemia/reperfusion. These, together with the diverse mechanisms that have been reported for TRPC channel activation under pathological conditions, argue for the need of more careful and systematic examinations of TRPC channels in ischemic brain damage.

In practice, a stroke therapy is more useful in post-stroke than during or pre-stroke application since in most cases, the need for treatment is only realized after the stroke has occurred. Therefore, a better understanding of mechanisms that contribute to neuronal death during reperfusion and/or in ischemic penumbra instead of the core area, or any intervention that would facilitate the recovery of damaged neurons, if possible, from the death pathway will be desirable for therapeutic development. However, despite all the studies that have been discussed above, nothing is available on whether any TRPC channel would be a good target of post-stroke neuroprotection. Our *in vitro* experiment (Figure 4) suggests that it might be possible for the TRPC4/C5 inhibitors to have such a function, even though it might not be a complete protection. It may be common that targeting a single pathway or molecule post-stroke or post-OGD will not completely protect all neurons from ischemic injury, since even if the targeting is effective and represents the most critical one, irreversible damages that have occurred during ischemia will unlikely be rescued. Recently, the TRPC channel field has experienced an explosion of newly identified small molecular probes that can act as either selective agonists or antagonists of specific TRPC channel subtypes (Wang et al., 2020). This gives an opportunity to test whether selectively enhancing or suppressing the function of a specific TRPC subtype at different time points post MCAO will exacerbate or inhibit brain damage and neurological deficits. Of course, these experiments will need to be followed by cell type specific manipulation of TRPC channel function or expression and other approaches in order to gain mechanistic insights on how the channel contributes to the delayed neuronal death.

Mechanistically, even though Ca^2+^ signal is an obvious downstream event of TRPC channel activation, there can be many different kinds of consequences which may or may not be related to the Ca^2+^ signal. The downstream targets of Ca^2+^ also remain largely undefined, although both CaMKII and calcineurin have been implicated. Ca^2+^-dependent kinases could also include those that phosphorylate NMDA receptor NR2B subunits, e.g., DAPK1, which is known to play a role in stroke (Nair et al., 2013). TRPC channels can mediate DAPK1 activation through calcineurin or calmodulin (Shamloo et al., 2005; Nair et al., 2013). Also, as has been illustrated for TRPC6, calpain is another possible candidate of Ca^2+^ regulation and it is known to play a role in NMDA receptor-mediated cell death (Lankiewicz et al., 2000; Simpkins et al., 2003). Therefore, specific inhibitors of the above Ca^2+^-regulated enzymes, calpain, CaMKII, calcineurin, and DAPK1 may be used to dissect their roles.

In summary, although TRPC channels appear to be ideally suited for integrating mGluR signaling to contribute to excitotoxicity to neurons under conditions of ischemic stroke, critical data demonstrating their involvement in neuronal damage or protection are only beginning to emerge. The pathological significance and roles of TRPC channels in ischemia/reperfusion remain to be fully elucidated. More detailed studies on regulatory mechanisms of TRPC channels will also shed lights on their functional significance in stroke pathology and future drug development.

## Methods

### Animals

All animal procedures were carried out in accordance with the NIH guidelines for the Care and Use of Laboratory Animals and approved by the Animal Welfare Committee of the University of Texas Health Science Center at Houston. TRPC1 KO (*Trpc1*^–/–^) and quadruple TRPC1, C4, C5, C6 KO (QuadKO) mice (129/SvEv and C57BL/6J mixed background) were created and maintained as described (Dietrich et al., 2007; Formoso et al., 2020); TRPC4 KO (*Trpc4*^–/–^) in C57BL/6 background were generated and maintained as previously described (Freichel et al., 2001).

### Mouse Ischemic Models

Focal ischemia (intraluminal suture) model of MCAO was carried out for 40 min followed by reperfusion as previously described (Sun et al., 2019). Animals were male QuadKO mice (8–9 weeks, ∼25 g) and age-matched wild type controls (C57BL/6J). In the MCA\CCAO model, unilateral occlusion of the left middle cerebral artery (MCA) and the left common carotid artery (CCA) was made as previously described (Aronowski et al., 1994; Zhao et al., 2015) using male wild type C57BL/6J and TRPC4 KO mice (8 weeks old). The occlusion times were 30, 90, and 120 min followed by reperfusion. With this method, the infarction is limited to the cortical tissue.

### Oxygen and Glucose Deprivation (OGD) Models

Primary cultures of cortical neurons were established using postnatal day 0 mouse pups (M/F) according to the description (Facci and Skaper, 2012). Cortical tissues from wild type (C57BL/6J), TRPC1 KO, TRPC4 KO, and QuadKO pups were triturated in a papain dissociation solution (Worthington) supplemented with DNase (200 units/ml) and incubated for 1 h in 37°C CO_2_ incubator. After stopping the reaction by adding ovomucoid (10 mg/ml), DNase (200 units/ml), and 10% fetal bovine serum in Earle’s balanced salt solution, cells were plated at 2 × 10^5^ per well in 500 μl of neurobasal medium supplemented with B27 (Invitrogen) and Glutamax (invitrogen) on poly-D-lysine (Sigma)-coated 12-mm round coverslips in 24-well culture plates. Another 500 μl of medium with arabinofuranosyl cytidine (1 μM final) was then added, and 20–30% of the medium was exchanged every other day until the cells were used for experiments (usually 16–18 days). For OGD, the medium was replaced with the deoxygenated glucose-free bicarbonate solution and the plate transferred to an anaerobic incubator containing 5% CO_2_ and 94% N_2_ (1% O_2_) atmosphere for 2 h. After OGD, the cells were rinsed with oxygenated glucose (20 mM)-containing bicarbonate solution, fed with the original neurobasal medium, and cultured for 0 or 24 h at 37°C, 5% CO_2_ (Ying et al., 1997).

Acute brain slices were prepared from wild type and TRPC4 KO mice (M, P15) as previously described (Tian et al., 2014a). Coronal slices (350 μm) were incubated in normal artificial cerebrospinal fluid (aCSF) consisting of (in mM): 125 NaCl, 26 NaHCO_3_, 1 MgSO_4_, 2.5 KCl, 1.25 NaH_2_PO_4_, 2 CaCl_2_, and 10 glucose, bubbled with 95% O_2_/5% CO_2_ at 35°C for at least 90 min before experiments. To induce OGD, slices were transferred to 15 ml conical tubes containing aCSF without glucose, which were bubbled with N_2_/CO_2_ at 35°C for 30 min. After the OGD treatment, the slices were returned to the glucose containing aCSF bubbled with 95% O_2_/5% CO_2_ at 35°C for 3 h.

## Data Availability Statement

The original contributions generated for this study are included in the article/supplementary materials, further inquiries can be directed to the corresponding author.

## Ethics Statement

The animal study was reviewed and approved by Animal Welfare Committee of University of Texas Health Science Center at Houston.

## Author Contributions

JJ prepared illustrations and wrote the manuscript. MZ wrote the manuscript. All authors proofread and approved the manuscript.

## Conflict of Interest

The authors declare that the research was conducted in the absence of any commercial or financial relationships that could be construed as a potential conflict of interest.
